# Evidence for sex-specific genetic architectures across a spectrum of human complex traits

**DOI:** 10.1186/s13059-016-1025-x

**Published:** 2016-07-29

**Authors:** Konrad Rawlik, Oriol Canela-Xandri, Albert Tenesa

**Affiliations:** 1 The Roslin Institute, Royal (Dick) School of Veterinary Studies, The University of Edinburgh, Easter Bush Campus, Midlothian, EH25 9RG Scotland UK; 2 MRC Human Genetics Unit at the MRC IGMM, University of Edinburgh, Western General Hospital, Crewe Road South, Edinburgh, EH4 2XU UK

**Keywords:** Gene-by-sex interactions, Sex-specific genetic architecture, Genomic prediction

## Abstract

**Background:**

Sex differences are a common feature of human traits; however, the role sex determination plays in human genetic variation remains unclear. The presence of gene-by-sex (GxS) interactions implies that trait genetic architecture differs between men and women. Here, we show that GxS interactions and genetic heterogeneity among sexes are small but common features of a range of high-level complex traits.

**Results:**

We analyzed 19 complex traits measured in 54,040 unrelated men and 59,820 unrelated women from the UK Biobank cohort to estimate autosomal genetic correlations and heritability differences between men and women. For 13 of the 19 traits examined, there is evidence that the trait measured is genetically different between males and females. We find that estimates of genetic correlations, based on ~114,000 unrelated individuals and ~19,000 related individuals from the same cohort, are largely consistent. Genetic predictors using a sex-specific model that incorporated GxS interactions led to a relative improvement of up to 4 % (mean 1.4 % across all relevant phenotypes) over those provided by a sex-agnostic model. This further supports the hypothesis of the presence of sexual genetic heterogeneity across high-level phenotypes.

**Conclusions:**

The sex-specific environment seems to play a role in changing genotype expression across a range of human complex traits. Further studies of GxS interactions for high-level human traits may shed light on the molecular mechanisms that lead to biological differences between men and women. However, this may be a challenging endeavour due to the likely small effects of the interactions at individual loci.

**Electronic supplementary material:**

The online version of this article (doi:10.1186/s13059-016-1025-x) contains supplementary material, which is available to authorized users.

## Background

Phenotypic differences between men and women are a pervasive feature of quantitative traits. Sex provides two different environmental contexts determined by the hormonal milieu, differential gene expression between the sexes [1, 2], and lifetime systematic differences in general environmental exposures arising, for instance, as a consequence of different gender roles in society. This raises the possibility of sex-specific autosomal genetic effects, induced by gene–environment interactions, and differences in heritability among sexes that contribute to inter-sex phenotypic variation [3–8].

Previous studies have used pedigrees to show that heritability differs between the sexes for a range of, mainly, low-level phenotypes [3]. However, to what extent differences observed in low-level phenotypes affect high-level complex traits and whether such differences can be observed in high-level complex traits remains unclear. Furthermore, differences in heritability do not imply differences in genetic architecture as they could arise as a consequence of differences in environmental variances between the sexes. It is important, therefore, to further examine differences in genetic effects directly or by estimating genetic correlations between sexes.

Studies of high-level complex traits which have examined differences in genetic effects between sexes have, however, been largely restricted to various anthropometric traits. Although familial studies have repeatedly reported differences between the genetic architecture for these phenotypes [4, 9], such findings have contrasted with studies on cohorts of unrelated individuals which have failed to find significant differences in genetic effects [5, 6, 8] or to identify significantly associated sex-specific single-nucleotide polymorphisms (SNPs) for traits such as height and body mass index (BMI) [5, 10, 11]. These differences could potentially arise due to biases in familial estimates due to shared environmental variance, differences in phenotype definition between different studies, or simply lack of power. To understand the nature of such discrepancies, it is important that estimations of sex genetic heterogeneity from related and unrelated individuals are made using large numbers of individuals of the same population and a uniform definition of phenotype.

To assess the extent of gene-by-sex (GxS) interactions in a human population, we tested for differences in genetic effects between men and women across 19 high-level complex traits. Specifically, we demonstrate the presence of GxS interactions by estimating both sex-specific heritabilities and genetic correlations between sexes using individual-level SNP data from ~114,000 unrelated and ~19,000 related individuals genotyped for up to 525,242 SNPs. Finally, we provide further evidence that supports the hypothesis that sex-determined genetic heterogeneity is present in high-level phenotypes by demonstrating that the observed GxS interactions can be utilised in practice to improve prediction of phenotypes based on genotypic information.

## Results and discussion

### Overview

To provide a broad overview of sex-specific genetic architecture in a human population we examined the presence of GxS interactions and sex-specific heritabilities across a broad spectrum of quantitative traits in ~114,000 unrelated white British participants in the UK Biobank [12] (Additional file 1: Table S1) who had been genotyped for 319,038 common autosomal SNPs (minor allele frequency (MAF) >5 %; see “Methods”). The 19 phenotypes considered were height, BMI, waist circumference (WC), hip circumference (HC), waist to hip ratio (WHR), body fat percentage (BF%), basal metabolic rate (BMR), age at completion of full time education for individuals without university education (education age), fluid intelligence score (FI score), a cognitive function score (CF score), lifetime reproductive success (LRS), diastolic and systolic blood pressure (BP_dia_ and BP_sys_), peak expiratory flow (PEF), forced expiratory volume in 1 s (FEV_1_), forced vital capacity (FVC), ratio of FEV_1_ over FVC (FEV_1_/FVC), self-assessed overall health (overall health) and extent of cigarette smoking as measured in pack years (Pack years). Education age in the UK Biobank has only been recorded for individuals without university education and care has to be taken in the interpretation of results for this phenotype and comparisons with other studies which use duration of education as a measure for educational attainment. Twelve of these phenotypes showed pronounced differences in distribution between the sexes (Additional file 1: Table S2 and Figure S1).

We evaluated the sex-specific genetic architecture of these traits by modelling male and female observations as occurrences of a phenotype in two different environmental contexts. The model used includes a genetic correlation between the two instances of the phenotype which may differ from one, thus providing evidence for a non-proportional change in the genetic effects between the two [13]. At the same time we allow for differences in heritability between the two sexes, which, on the other hand, can provide evidence for proportional changes in genetic effects or differences in environmental influences. Specifically, we fitted a bivariate linear mixed model (LMM) using the DISSECT software [14] (see “Methods”). The model included independent genetic and residual variances for male and female phenotypes and a genetic correlation. We tested whether genetic correlations were significantly different from one or whether heritabilities differed between men and women separately using likelihood ratio tests.

### Sex-specific heritability

**Fig. 1:**
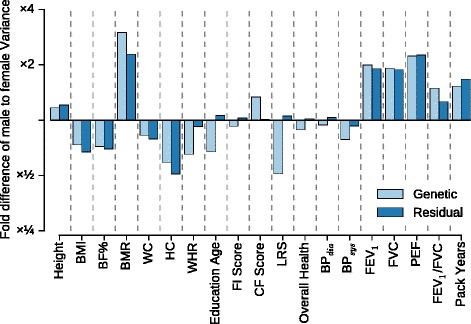
Differences in variance components between the sexes. The fold difference between male and female genetic and residual variance components as estimated using common SNPs in unrelated white British individuals. Values larger than one indicate a larger variance in males, values smaller than one a larger variance in females

Seven traits showed significant differences (*P* < 0.05) in heritability (Table 1). In addition, the blood pressure traits (BP_dia_ and BP_sys_) showed more pronounced differences in heritability when individuals with hypertension or taking blood pressure medication were included in the analysis, whilst adjusting for these factors (Additional file 1: Table S3). These differences may arise as a consequence of different environmental contexts, different amounts of genetic variation, or both. However, for five of the seven phenotypes for which we detected a significant difference in heritability, these differences could be explained by larger differences in genetic, rather than residual, variance between the two sexes (Fig. 1). Moreover, in general, larger genetic variances in one sex were observed together with larger residual variance components in the same sex. The exceptions were education age, FI score, LRS, overall health, and BP_dia_, for which the relationship between the sexes with regard to the size of genetic and residual variance was reversed; that is, we find, for example, that the genetic variance for LRS is larger in women while the residual variance for this trait is larger in men. With the exception of HC, all traits which show significant differences in heritability (BMR, WHR, education age, CF score, LRS, and FEV_1_/FVC) were found to have larger differences between the sexes for genetic rather than residual variances. Hence, overall the results support the view that differences in heritability are a consequence of a difference in genetic architecture or gene–environment interactions associated with sex rather than arising purely due to a significant difference in environmental variance.

**Table 1:** Estimates of sex-specific heritabilities and genetic correlations in unrelated white British individuals

	*h*_*m*_^2^		*h*_*f*_^2^		*r*_*g*_		*P* value	
	Est.	S.E.	Est.	S.E.	Est.	S.E.	*r*_*g*_ ≠ 1	*h*_*m*_^2^ ≠ *h*_*f*_^2^
Height	0.53	0.007	0.54	0.007	0.96	0.01	3 × 10^-5^	0.3
BMI	0.27	0.008	0.26	0.007	0.95	0.02	0.008	0.1
BF%	0.27	0.008	0.26	0.008	0.94	0.02	0.009	0.6
BMR	0.36	0.008	0.30	0.008	0.92	0.02	3 × 10^-5^	3 × 10^-8^
WC	0.24	0.008	0.23	0.008	0.90	0.03	0.0003	0.5
HC	0.27	0.008	0.24	0.008	0.88	0.02	2 × 10^-6^	0.01
WHR	0.19	0.008	0.25	0.008	0.76	0.03	9 × 10^-14^	1 × 10^-7^
Education age	0.08	0.011	0.13	0.010	0.92	0.09	0.2	0.004
FI score	0.28	0.022	0.27	0.020	0.93	0.06	0.1	0.8
CF score	0.12	0.008	0.09	0.007	0.81	0.06	0.0008	0.02
LRS	0.04	0.014	0.08	0.009	0.56	0.17	0.03	0.03
Overall health	0.12	0.008	0.12	0.007	0.97	0.05	0.3	0.6
BP_dia_	0.18	0.011	0.20	0.009	0.87	0.04	0.001	0.3
BP_sys_	0.17	0.011	0.20	0.009	0.88	0.05	0.009	0.1
FEV_1_	0.28	0.010	0.27	0.010	0.92	0.03	0.004	0.5
FVC	0.26	0.010	0.26	0.009	0.95	0.03	0.06	0.8
PEF	0.23	0.010	0.23	0.009	0.93	0.03	0.02	0.9
FEV_1_/FVC	0.29	0.010	0.25	0.009	0.97	0.03	0.1	0.01
Pack years	0.17	0.012	0.19	0.012	0.90	0.06	0.05	0.5

*h*_*m*_^2^, *h*_*f*_^2^ are proportions of phenotypic variance explained by common autosomal SNPs for males and females, respectively. *P value* indicates *p* values from likelihood ratio test against constrained models with *r*_*g*_ = 1 and *h*_*m*_^2^ = *h*_*f*_^2^, respectively

*Est.* estimate, *S.E.* standard error

### Genetic correlations between sexes

For 13 out of the 19 traits studied we found evidence of GxS interactions because the genetic correlation (r_g_) between the traits measured in men and women was significantly different from one. Estimates of r_g_ for these phenotypes ranged from 0.96 for height to 0.56 for LRS, with six of the phenotypes having an estimated r_g_ below 0.9. Importantly GxS interactions were found across all categories of phenotypes, including anthropometric, cognitive, pulmonary, and cardiovascular. Familial studies [9] have reported evidence supporting GxS interactions across a range of anthropometric phenotypes. However, whilst genome-wide association studies have identified SNP-by-sex interactions for some anthropometric traits like WHR [5, 8], identifying these has proven to be extremely challenging, raising the question of whether the expected interactions exist. Similarly to familial studies, we find evidence of GxS interactions in all the anthropometric traits studied using unrelated samples of the population, albeit our estimates of r_g_ are generally higher than those reported in twin studies [4]. Previous analyses on unrelated individuals for either height or BMI [5, 6, 8] did not find significant differences in genetic effects, suggesting these studies lacked power due to smaller samples sizes or the methodology used. In particular, our results are consistent with the standard errors of Yang et al. [6], who, using a sample size of individual-level data less than half of that used here, did not obtain significant results for either height or BMI using the same methodology.

Beyond anthropometric traits, the low genetic correlation for LRS among sexes is of particular interest due to its implications in, for instance, maintaining genetic variation in the population. A GxS interaction for LRS suggests that the genetic determinants that contribute to the reproductive fitness of men and women may be different or may have different effects, which could play an important role in maintaining genetic variation in the population [15]. In addition, the differences in heritabilities between men and women, which are a consequence of women having twice as much genetic variation for this trait as men, suggest that genetics plays a larger role in the reproductive fitness of women than men.

### Effects of study population

**Fig. 2:**
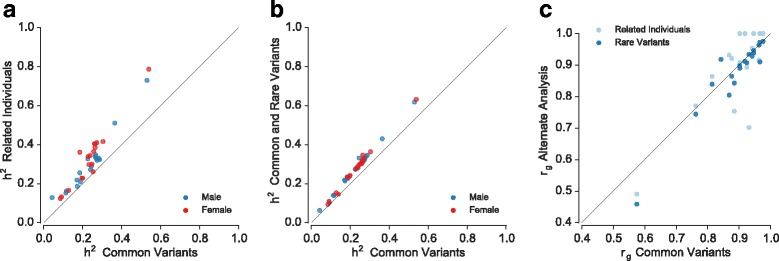
Effect of relatedness and SNP density on estimates of heritability and genetic correlations. Estimates of male and female heritability obtained using common variants in unrelated individuals against those obtained using **a** related individuals and common variants or **b** including common and rare variants for unrelated individuals. **c** Comparison of estimates of genetic correlations between these two alternative analyses with the estimates obtained on the main set of unrelated individuals with common variants

To confirm the robustness of our results we performed several additional analyses. First, we re-estimated variance components for the same set of unrelated individuals including rare variants (MAF 0.37–5 %) together with common variants, that is, all available SNPs that passed our quality control (Additional file 1: Figure S2). Second, we performed the analyses, using common variants, on a set of ~19,000 related white British participants which partially overlaps (10,112 individuals) with the unrelated cohort. The results of these analyses do not represent an independent replication but a way of assessing the effect of the changed tagging structure due to long-range linkage disequilibrium and shared environment in these related individuals. In line with expectations [16, 17] the estimates of heritability increased significantly in both alternative analyses (Fig. 2a), whilst estimates of r_g_ remained largely unaffected (Fig. 2b; Additional file 1: Table S4). The correlation between r_g_estimates based on common variants and combined common and rare variants in unrelated individuals was 0.98, whilst between related and unrelated individuals was 0.79 (Fig. 2c).

As four of the phenotypes we considered (education age, FI score, LRS, and overall health) were categorical in nature, we investigated to what extent our results, based on estimates obtained on the observed scale, could be affected by differences in phenotypic distribution between the sexes. To this end we repeated the analysis using common variants on a random subset of the unrelated white British cohort, sampled so as to ensure that phenotypic distributions for both sexes are equal (see “Methods”). The results of this analysis are consistent with those obtained on the whole cohort (Additional file 1: Table S5), suggesting that the observed differences in heritability on the observed scale are not likely to be driven by differences in phenotype distribution in the two sexes. Similarly, to confirm that differences in phenotypic distributions of the considered quantitative traits did not lead to spurious genetic correlations below one, we performed an additional analysis on rank normalized phenotypes (Additional file 1: Additional methods and results). The results were highly consistent with those reported here for untransformed phenotypes (Additional file 1: Figure S3 and Tables S6 and S7). Additionally we performed a simulation study (Additional file 1: Additional methods and results) of traits with differing phenotypic distributions and heritabilities but genetic correlations of one, which further supports our view that these factors do not lead to spurious results (Additional file 1: Table S8 and Figure S4).

Finally, we investigated three other potential sources of bias: the presence of spouses in the UK Biobank cohort [18], differences in socio-economic status among the sampled male and female population, and sex differences in overall health status. In particular, the latter could potentially bias our results due to the possibility of differences in the enrolment of the male and female components of the study population [19]. To investigate the effect of couples, we excluded one individual at random from each genotyped spouse pair and repeated all the main analyses. Despite the reduction in sample size, the results of these analyses were very similar to the main results (Additional file 1: Figure S5, Tables S6 and S9). Likewise, we repeated all the main analyses adjusting for additional factors related to socioeconomic status and health (that is, self-reported overall health status, Townsend Deprivation Index and educational attainment). These results were also highly consistent with the results of the main analysis (Additional file 1: Figure S6, Tables S6 and S10), suggesting that our results are not substantially influenced by these socio-economic and health status factors.

### Sex-specific genomic prediction

An alternative way of testing whether the genetic architecture of the two sexes is different or whether there are GxS interactions is to perform genomic predictions under a model that accounts for these interactions (that is, a sex-specific model) and another that does not (that is, a sex-agnostic model). To this end, we estimated separate male and female common SNP effects in the sex-specific bivariate model as well as in the sex-agnostic univariate model [20] (see “Methods”). We used our ~114,000 unrelated white British individuals as the training population and predicted additive genetic values for a separate cohort of ~12,000 genotyped UK Biobank individuals who self-reported as white British but had failed to be confirmed as such by the principal components analysis (PCA; see “Methods”). Prediction accuracy was measured as the correlation between predicted additive genetic value and observed phenotypes adjusted for fixed effects. The sex-specific model outperformed the sex-agnostic model for a majority of phenotypes, i.e., 14 out of 19 (Additional file 1: Table S11). In particular, considering only the ten phenotypes with evidence of genetic heterogeneity between sexes (*P* > 0.05 for r_g_ ≠ 1 or *h*_*m*_^2^ ≠ *h*_*f*_^2^) and substantial heritability (*h*_*m*_^2^ > 0.2 and *h*_*f*_^2^ > 0.2), all but one (WC) showed an improvement in prediction accuracy, with BMR showing the largest improvement (>4 %) and with the mean improvement across these traits being 1.4 %. These results add further evidence to the presence of sex-specific genetic effects and, since sex could be considered as a surrogate measure of different environmental factors, provide evidence that the utilisation of gene–environment interactions can improve the accuracy of genomic profiling.

## Conclusions

Our analyses of a large cohort using individual-level genotype data provide a broad assessment of differences in genetic architecture between sexes and shows that contributions of sex-specific genetic effects, although of modest magnitude, may be found across a broad spectrum of traits. While significance does not imply that these effects are large, we are able to reproduce our results, whilst simultaneously quantifying the impact of these effects, using genomic predictions in an independent cohort. Taking WHR as an example, 291 associations are reported in the GWAS Catalog [21], which may be taken as a lower bound of the total number of associated variants. We may then observe that, based on the assumption that these contribute equally to prediction accuracy in the univariate model, differences in genetic architecture between the sexes are equivalent to a lower bound of about seven additional associated variants.

The general lack of significant SNP-by-sex interactions in genome-wide association studies suggests that these effects may be a consequence of the accumulated effect of many interactions of small effect, identification of which may require even larger sample sizes than used here. Further research to identify the causes that determine sex genetic heterogeneity will need to disentangle whether sex genetic heterogeneity may arise as a consequence of interactions with genetic loci located on the sex chromosomes, differences in gene control due to differences in the sex-specific cellular environment, or more general differences in environmental exposures between the sexes. Furthermore, we demonstrate, using sex as a surrogate measure of environmental exposure, how to incorporate gene-by-environment interactions into genomic prediction models.

## Methods

### Genotype quality control

For our analysis, we used the data for the individuals genotyped in phase 1 of the UK Biobank genotyping program; 49,979 individuals were genotyped using the Affymetrix UK BiLEVE Axiom array and 102,750 individuals using the Affymetrix UK Biobank Axiom array. Details regarding genotyping procedure and genotype calling protocols are provided elsewhere (http://biobank.ctsu.ox.ac.uk/crystal/refer.cgi?id=155580). We performed quality control using the entire set of genotyped individuals before extracting the white British cohort used in our analysis. From the overlapping markers, we excluded those which are multi-allelic and those whose overall missingness rate exceeded 2 % or which exhibited a strong platform specific missingness bias (Fisher’s exact test, *P* < 10^-100^). We also excluded individuals if they exhibited excess heterozygosity, as identified by UK Biobank internal quality control procedures (http://biobank.ctsu.ox.ac.uk/crystal/refer.cgi?id=155580), if their missingness rate exceeded 5 %, or if their self-reported sex did not match genetic sex estimated from X chromosome inbreeding coefficients. These criteria resulted in a reduced dataset of 151,532 individuals. Finally, we only kept variants that did not exhibit departure from Hardy–Weinberg equilibrium (*P* < 10^-50^) in the unrelated (subset of individuals with a relatedness below 0.0625) white British subset of the cohort. To define the white British cohort, we performed a PCA of all individuals passing genotypic quality control using a linkage disequilibrium pruned set of 99,101 autosomal markers (http://biobank.ctsu.ox.ac.uk/crystal/refer.cgi?id=149744) that passed our SNP quality control protocol. The white British individuals were defined as those for whom the projections onto the leading 20 genomic principal components fell within three standard deviations of the mean and who self-reported their ethnicity as white British. Those individuals who self-reported as white British but who were excluded based on the PCA analysis formed the test white British sample used in prediction. We furthermore pruned the set of white British individuals, removing one individual from pairs with relatedness above 0.0625 (corresponding to second degree cousins) to obtain a datasets of unrelated confirmed white British individuals.

### Phenotype quality control

We obtained measures for waist circumference (UKBID 48), hip circumference (UKBID 49), standing height (UKBID 50), BMI (UKBID 21001), body fat percentage (UKBID 23099), basal metabolic rate (UKBID 23105), self-reported age of completion of full-time education for individuals without university education (UKBID 845), number of offspring (UKBID 2405 and 2734), fluid intelligence score (UKBID 20016), diastolic blood pressure (UKBID 4079), systolic blood pressure (UKBID 4080), forced volume vital capacity (UKBID 3062), forced expiratory volume in 1 s (UKBID 3063), peak expiratory flow (UKBID 3064), and self-reported overall health (UKBID 2178). Further information about these, including details of measurement protocols, can be accessed through the UK Biobank resource (http://biobank.ctsu.ox.ac.uk/crystal/index.cgi) using the provided UKBID. We additionally computed several derived phenotypes based on information contained in the UK Biobank. Specifically, we computed WHR and FEV_1_/FVC as ratios of WC, HC, FEV_1_ and FVC, respectively, and furthermore rescaled BMR to have a standard deviation of 1 in the population due to numerical problems in model fitting on the measurement scale. LRS was calculated as the self-reported number of offspring for individuals who have completed their reproductive life. These were defined as men aged over 60 years and women who reported either having had their menopause or undergone a hysterectomy or who were aged over 60 years. We furthermore excluded individuals who reported having had in excess of 15 offspring. We constructed a cognitive function score (CF score) as the first principal component of several cognitive measures, specifically the results of a reaction time test (UKBID 20023), time to complete, and number of incorrect guesses during completion of a pairs matching test (UKBID 400 and 399). Prior to PCA we excluded individuals who were more than 5 standard deviations from the population for any of these measures. The number of pack years of smoking was calculated based on smoking history information as described elsewhere (http://biobank.ctsu.ox.ac.uk/crystal/field.cgi?id=20161). Self-reported overall health status was measured as the answer to the question “In general how would you rate your overall health?” excluding “Do not know”/“Prefer not to answer” we coded the possible four answers as numerical values 1 (“Excellent”) to 4 (“Poor”) and fitted all models on this observed scale. For the cardiovascular phenotypes (BP_dia_ and BP_sys_) we excluded all individuals who reported taking blood pressure medication (UKBID 6153 and 6177). We removed outliers from WC, HC, height, BMI, BF%, BMR, WHR, education age, BP_dia_, BP_sys_, FVC, FEV_1_, PEF, and FEV_1_/FVC, defining outliers as males and females who were outside ±3 standard deviations from their gender mean of all the individuals in the white British cohort.

### Estimation of heritability and genetic correlations

We used a linear mixed model approach [22] to estimate sex-specific variance components and genetic correlations between the sexes. Specifically, we computed restricted maximum likelihood (REML) fits for the bivariate model:

$$ \mathbf{y}=\left(\begin{array}{c}\hfill {\mathbf{y}}_{\boldsymbol{m}}\hfill \\ {}\hfill {\mathbf{y}}_{\boldsymbol{f}}\hfill \end{array}\right)=\left(\begin{array}{cc}\hfill {\mathbf{x}}_{\boldsymbol{m}}\hfill & \hfill \mathbf{0}\hfill \\ {}\hfill \mathbf{0}\hfill & \hfill {\mathbf{x}}_{\boldsymbol{f}}\hfill \end{array}\right)\left(\begin{array}{c}\hfill {\boldsymbol{\upbeta}}_{\boldsymbol{m}}\hfill \\ {}\hfill {\boldsymbol{\upbeta}}_{\boldsymbol{f}}\hfill \end{array}\right)+\left(\begin{array}{cc}\hfill {\mathbf{G}}_{\boldsymbol{m}}\hfill & \hfill \mathbf{0}\hfill \\ {}\hfill \mathbf{0}\hfill & \hfill {\mathbf{G}}_{\boldsymbol{f}}\hfill \end{array}\right)\left(\begin{array}{c}\hfill {\mathbf{a}}_{\boldsymbol{m}}\hfill \\ {}\hfill {\mathbf{a}}_{\boldsymbol{f}}\hfill \end{array}\right)+\left(\begin{array}{c}\hfill {\mathbf{e}}_{\boldsymbol{m}}\hfill \\ {}\hfill {\mathbf{e}}_{\boldsymbol{f}}\hfill \end{array}\right), $$

where for sex *x*, X_*x*_ is the incidence matrix for fixed effects β_*x*_, including a constant column modeling the mean, G_*x*_ is the matrix of standardized genotypes, a_*x*_ is the vector of SNP effects, and e_*x*_ is the vector of residuals. Priors were placed on the SNP effects and residuals:

$$ \left(\begin{array}{c}\hfill {\mathbf{a}}_{\boldsymbol{m}}\hfill \\ {}\hfill {\mathbf{a}}_{\boldsymbol{f}}\hfill \end{array}\right)\sim N\left(\left(\begin{array}{c}\hfill \mathbf{0}\hfill \\ {}\hfill \mathbf{0}\hfill \end{array}\right),\left(\begin{array}{cc}\hfill {\sigma}_{{\text{\textit{\textsf{g}}}}_m}^2\hfill & \hfill \rho \sqrt{\sigma_{{\text{\textit{\textsf{g}}}}_m}^2{\sigma}_{{\text{\textit{\textsf{g}}}}_f}^2}\hfill \\ {}\hfill \rho \sqrt{\sigma_{{\text{\textit{\textsf{g}}}}_m}^2{\sigma}_{g_f}^2}\hfill & \hfill {\sigma}_{{\text{\textit{\textsf{g}}}}_f}^2\hfill \end{array}\right)\otimes \mathrm{I}\right)\kern0.5em \mathrm{and}\kern0.5em \left(\begin{array}{c}\hfill {\mathbf{e}}_{\boldsymbol{m}}\hfill \\ {}\hfill {\mathbf{e}}_{\boldsymbol{f}}\hfill \end{array}\right)\sim N\left(\left(\begin{array}{c}\hfill \mathbf{0}\hfill \\ {}\hfill \mathbf{0}\hfill \end{array}\right),\left(\begin{array}{cc}\hfill {\sigma}_{e_m}^2\hfill & \hfill \mathbf{0}\hfill \\ {}\hfill \mathbf{0}\hfill & \hfill {\sigma}_{e_f}^2\hfill \end{array}\right)\otimes \mathrm{I}\right) $$

where *N*(μ,Σ) is the multivariate normal distribution with mean μ and covariance Σ, I is the identity matrix, and ⊗ is the Kronecker product between matrices. Using the estimates of the model parameters, heritabilities for each sex were computed as ${h}_x^2=\frac{\sigma_{{\mathrm{g}}_x}^2}{\sigma_{g_x}^2+{\sigma}_{e_x}^2}$, with the reported confidence intervals calculated based on standard errors of the model parameters. We additionally obtained REML fits of the above model under the constraints *ρ* = 1 and $\frac{\sigma_{{\text{\textit{\textsf{g}}}}_m}^2}{\left({\sigma}_{{\text{\textit{\textsf{g}}}}_m}^2+{\sigma}_{e_m}^2\right)}=\frac{\sigma_{{\text{\textit{\textsf{g}}}}_f}^2}{\left({\sigma}_{{\text{\textit{\textsf{g}}}}_f}^2+{\sigma}_{e_f}^2\right)}$, respectively. For the latter we reparametrized the model in terms of parameters *σ*_*m*_^2^, *σ*_*f*_^2^ and λ, setting ${\sigma}_{{\text{\textit{\textsf{g}}}}_m}^2={\sigma}_m^2,{\sigma}_{{\text{\textit{\textsf{g}}}}_f}^2={\sigma}_f^2,{\sigma}_{e_m}^2=\lambda {\sigma}_m^2\kern0.5em \mathrm{and}\kern0.5em {\sigma}_{e_f}^2=\lambda {\sigma}_f^2$ and optimized the REML. Using these restricted models, we tested for genetic correlations between the sexes different from unity and unequal heritabilities for the two sexes using likelihood ratio tests using 1 degree of freedom. All analyses included the leading 20 genomic principal components as fixed effects in order to adjust for population structure. Furthermore, age was included in all analyses as a fixed effect, with the exception of LRS, where we included year of birth, which better captures cohort effects. Finally, analyses of pulmonary phenotypes included further fixed effects; specifically, both height and pack years were included for PEF and FEV_1_ and only pack years was included for FEV_1_/FVC. All models were fitted using the DISSECT software (http://www.dissect.ed.ac.uk) [14] on the UK National Supercomputer (ARCHER).

### Alternative analyses

Analyses performed to investigate robustness of the results utilized the following datasets. From the dataset of all individuals who were identified as white British, we extracted the set of individuals who had at least one other white British individual with a relatedness, as estimated based on common SNPs, above 0.0625. This cohort of 19,695 related white British individuals partially overlapped (i.e., 10,112 individuals overlapped) with the unrelated white British cohort used in the main analysis as, for the latter, only one of each pair of related individuals was excluded.

For the additional analysis of categorical phenotypes, we subsampled the set of unrelated white British individuals for each phenotype to maximize the total sample size whilst ensuring that the phenotypic distribution in the sexes was equal. To this end we stratified the individuals within each sex by the phenotype value and for each strata included all individuals of the sex with fewer samples and randomly sampled an equal number of individuals for the other sex.

### Genomic prediction

Predictions *ŷ*_*i*_ for a phenotype of individual *i* were computed as:

$$ {\widehat{y}}_i={\displaystyle \sum_{j=1}^M\frac{s_{ij}-{\mu}_j}{\sigma_j}}{a}_j, $$

where *s*_*ij*_ is the number of copies of the reference allele at SNP *j* for individual *i*, *M* is the total number of SNPs used for prediction, i.e., in our case the number of common SNPs, and *a*_*j*_ is the estimated SNP effect of SNP *j*, while *μ*_*j*_ and *σ*_*j*_ are the mean and standard deviation of the reference allele in the training population, i.e., the genotypically white British individuals. We estimated effects of all SNPs following either a standard univariate approach [20], i.e., fitting a LMM treating male and female phenotypes as one phenotype, or by estimating sex-specific SNP effects based on the bivariate model discussed previously. Specifically, SNP effects were estimated by their posterior mean with variance parameters and fixed effect parameters fixed to their REML estimates. The same fixed effect structure was used in both models, i.e., we included sex interactions in the fixed effects of the univariate model. Prediction accuracies were computed as the correlation between predicted phenotypes and observed phenotypes adjusted for fixed effects using estimated fixed effect coefficients.

## Additional file

Additional file 1: Additional methods and results. **Figure S1** Phenotypic distributions for both sexes for considered phenotypes. **Figure S2** SNP MAFs. **Figure S3** Comparison of the results of the main analysis with those obtained on rank normalized phenotypes. **Figure S4** Comparison of the results of the main analysis with those of simulated phenotypes. **Figure S5** Comparison of the results of the main analysis with those obtained after exclusion of spouses. **Figure S6** Comparison of the results of the main analysis with those obtained after adjusting for further socio-economic and health covariates. **Table S1** Phenotype-specific sample sizes for the sets of unrelated and related white British individuals. **Table S2** Sexual dimorphism indices for all phenotypes. **Table S3** Comparison of the parameter estimates for alternative analyses of blood pressure phenotypes. **Table S4** Estimates of heritabilities and genetic correlations for models fitted to related individuals with common SNPs and unrelated individuals including both common and common and rare SNPs. **Table S5** Estimates of heritabilities, genetic correlations and *P* values for differences in heritability and genetic correlations differing from one, for categorical phenotypes using subsamples of the unrelated white British individuals with matching phenotypic distributions between the sexes. **Table S6** Comparison of the results of the main analysis with those obtained on alternative analyses. **Table S7** Estimates of heritabilities, genetic correlations and *P* values for differences in heritability and genetic correlations differing from one, for rank normalized phenotypes. **Table S8** Parameter estimates for simulated phenotypes. **Table S9** Estimates of heritabilities, genetic correlations and *P* values for differences in heritability and genetic correlations differing from one, after exclusion of spouses. **Table S10** Estimates of heritabilities, genetic correlations and *P* values for differences in heritability and genetic correlations differing from one, when adjusting for additional socio-economic and health factors. **Table S11** Prediction accuracies for bivariate and univariate genomic prediction models for all traits. (DOCX 2206 kb)
