# Supplementary material for: Evidence for sex-specific genetic architectures across a spectrum of human complex traits
Source: Genome Biol. 2016 Jul 29;17:166. doi: 10.1186/s13059-016-1025-x (PMC4965887; doi:10.1186/s13059-016-1025-x)
Supplement: Additional file 1: — Additional methods and results. Figure S1 Phenotypic distributions for both sexes for considered phenotypes. Figure S2 SNP MAFs. Figure S3 Comparison of the results of the main analysis with those obtained on rank normalized phenotypes. Figure S4 Comparison of the results of the main analysis with those of simulated phenotypes. Figure S5 Comparison of the results of the main analysis with those obtained after exclusion of spouses. Figure S6 Comparison of the results of the main analysis with those obtained after adjusting for further socio-economic and health covariates. Table S1 Phenotype-specific sample sizes for the sets of unrelated and related white British individuals. Table S2 Sexual dimorphism indices for all phenotypes. Table S3 Comparison of the parameter estimates for alternative analyses of blood pressure phenotypes. Table S4 Estimates of heritabilities and genetic correlations for models fitted to related individuals with common SNPs and unrelated individuals including both common and common and rare SNPs. Table S5 Estimates of heritabilities, genetic correlations and P values for differences in heritability and genetic correlations differing from one, for categorical phenotypes using subsamples of the unrelated white British individuals with matching phenotypic distributions between the sexes. Table S6 Comparison of the results of the main analysis with those obtained on alternative analyses. Table S7 Estimates of heritabilities, genetic correlations and P values for differences in heritability and genetic correlations differing from one, for rank normalized phenotypes. Table S8 Parameter estimates for simulated phenotypes. Table S9 Estimates of heritabilities, genetic correlations and P values for differences in heritability and genetic correlations differing from one, after exclusion of spouses. Table S10 Estimates of heritabilities, genetic correlations and P values for differences in heritability and genetic correlations differing from one, when ad [file 13059_2016_1025_MOESM1_ESM.docx]

**Additional Methods and Results** *Normalised Phenotypes*

Phenotypic distributions differ significantly between the sexes for many of the considered phenotypes (Figure S1). While all models utilised include sex specific intercepts and variance components and hence are able to account for differences in phenotypic mean and variance, differences in higher order moments are not corrected. In order to investigate the impact that these higher order differences have on our results we repeated the analyses on the main cohort of unrelated genotypically White-British individuals using common variants for normalised phenotypes. Specifically we rank normalised all phenotypes within sex. That is we transformed phenotypes within each sex by mapping their normalised within sex rank to a standard normal distribution.

Parameter estimates obtained on normalised phenotypes did not differ substantially from those obtained on untransformed phenotypes (Figure S3). Similarly results for departure of genetic correlations from one and differential heritability between sexes are largely consistent with those obtained with the non-normalised phenotypes (Tables S6 and S7).

*Partner Correlations*

The UKBiobank cohort contains a number of spouse pairs [1] that may influence our results. To investigate whether and how the presence of partners could affect our results and conclusions we repeated our analysis using common variants on a subset of the main cohort of unrelated genotypically White-British individuals without spouse pairs. Specifically, for each spouse pair, identified using the methods of [1], we removed one partner choosing the sex of the dropped partner randomly with uniform probability for each pair.

Parameter estimates obtained on the subset of the cohort did not differ substantially from those obtained on the entire cohort (Figure S5). Similarly results for departure of genetic correlations from one and differential heritability between sexes are largely consistent with those obtained when spouse pairs are not included (Tables S6 and S9).

*Socio Economic Structure*

Ascertainment biases in socio-economic factors, sex distribution and overall health are likely to exist due to low recruitment rates. We assessed the impact of these factors within our data on our analysis by repeating the analyses using the main cohort of genotypically White-British individuals and common variants including additional covariates adjusting for socio-economic status, educational attainment and health. Specifically we included the Townsend Deprivation Index (UKBID 189), Educational Attainment (UKBID 6138) and Self-Reported Overall Health (UKBID 2178) as additional fixed effects. Townsend Deprivation Index was treated as a continuous covariate. Self-Reported Overall Health was fitted as a categorical covariate with four levels, while Educational attainment was fitted was fitted as a categorical covariate with one level for each combination of qualifications reported in the UK Biobank. Self-Reported Overall Health was not included in the analysis, when it was the focal phenotype.

Including these additional covariates lead to heritability estimates which were systematically lower compared to unadjusted estimates (Figure S6a), while no systematic change in estimates of genetic correlations was observed (Figure S6b). However the observed bias in heritability estimates applied to both sexes and results regarding differences in heritability between sexes were largely consistent with the main analysis (Tables S6 and S10). Using Education Age instead of Education Attainment as a covariate, despite the smaller sample size due to exclusion of individuals with University degrees in the former analysis, did not change the results substantially (data not shown).

*Blood Pressure Phenotypes*

Exclusion of individuals reporting taking blood pressure medication (see Methods) may inadvertently bias results, as prevalence of high blood pressure is higher amongst men than women. We there repeated the analyses for BP_sys_ and BP_dia_ in the main cohort of White British individuals on common variants including all individuals. Instead of excluding individuals we adjusted for blood pressure medication and hypertension status by including a categorical fixed effect with one level for each combination of self-reported hypertension status (extracted from UKBID 20002) and blood pressure medication status.

The results of this analyses are summarised in Table S3. Estimates of heritability were significantly lower, while estimates of genetic correlations between the sexes did not change significantly. Differences in heritability between the sexes became more pronounced and were significant for both BP_sys_ (P=0.008) and BP_dia_ (P=0.002).

*Simulations*

In order to investigate whether our results for genetic correlation could be spurious due to differences in phenotypic distributions and heritabilities in the sexes we followed a simulation approach, simulating ten phenotypes with $r_{g}=1$ and other parameters matched to those observed in some of the traits considered in the main analyses.

The simulations used genotypes of 114,264 genotypically white-british individuals from the UK Biobank (see Manuscript Methods). We simulated phenotypes with 5,000 QTLs, chosen uniformly at random, with a fixed rg of 1 but with separate heritabilities for males and females to mimic the range of observed heritabilities. Specifically we choose male heritabilities by equally spacing 10 values in the interval [0.047, 0.535] corresponding to the interval of heritability estimates obtained for the 19 traits in our main analysis. Female heritabilities were chosen by randomly sampling a heritability ratio from a normal distribution with variance matched to the empirical distribution of heritability ratios in our main analysis. We then transformed the male and female phenotypes to match the within sex means and variances observed for a randomly chosen phenotype from amongst the 19 traits considered in our main analysis. We then fitted models as described in the main Methods, removing the 5,000 QTLs for the corresponding phenotype from the genotypes used in model fitting (i.e., simulating the situation where causative variants are not genotyped).

These simulations did not yield any significant estimate of results genetic correlation significantly different from 1 (see Figure S4 and Table S8), further supporting our argument that differences in phenotypic means and variance components between the sexes do not lead to spurious results with regards to the estimates of genetic correlations we provide.

**References**

1. Tenesa A, Rawlik K, Navarro P, Canela-Xandri O: **Genetic determination of height-mediated mate choice.** *Genome Biol* 2016, **16:**269.

**Figure S1: Distributions of quantitative phenotypes considered.**

**
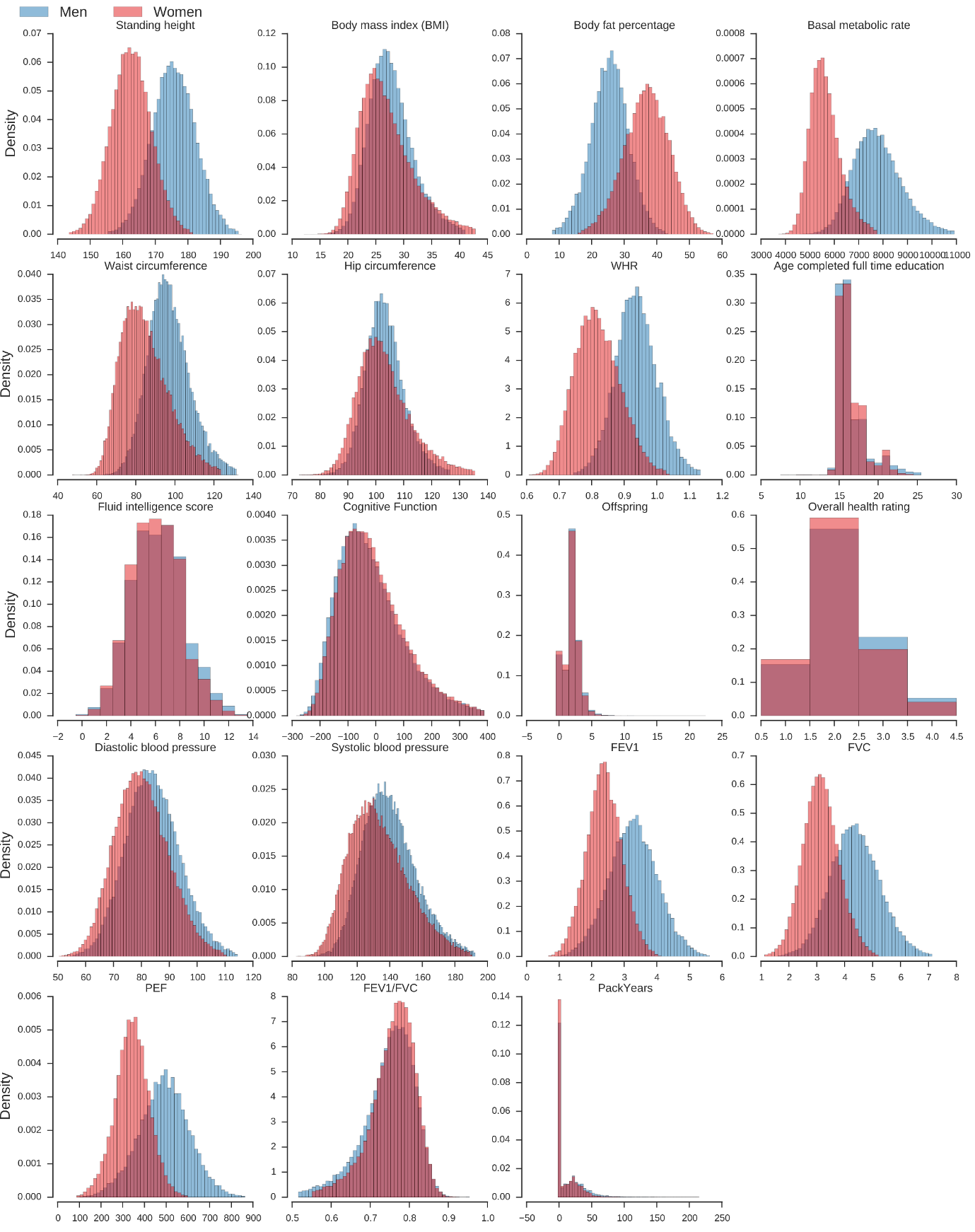
**

**Figure S2: Histogram of minor allele frequencies of used SNPS in the White-British cohort**


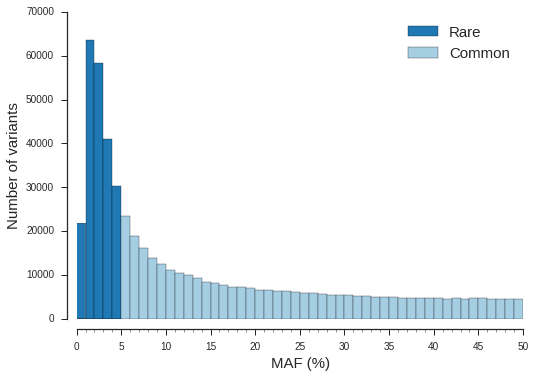


**Figure S3: Comparison of heritability and genetic correlation estimates between analyses using untransformed and rank normalised phenotypes on the main cohort of white-british individuals using common variants.**

**
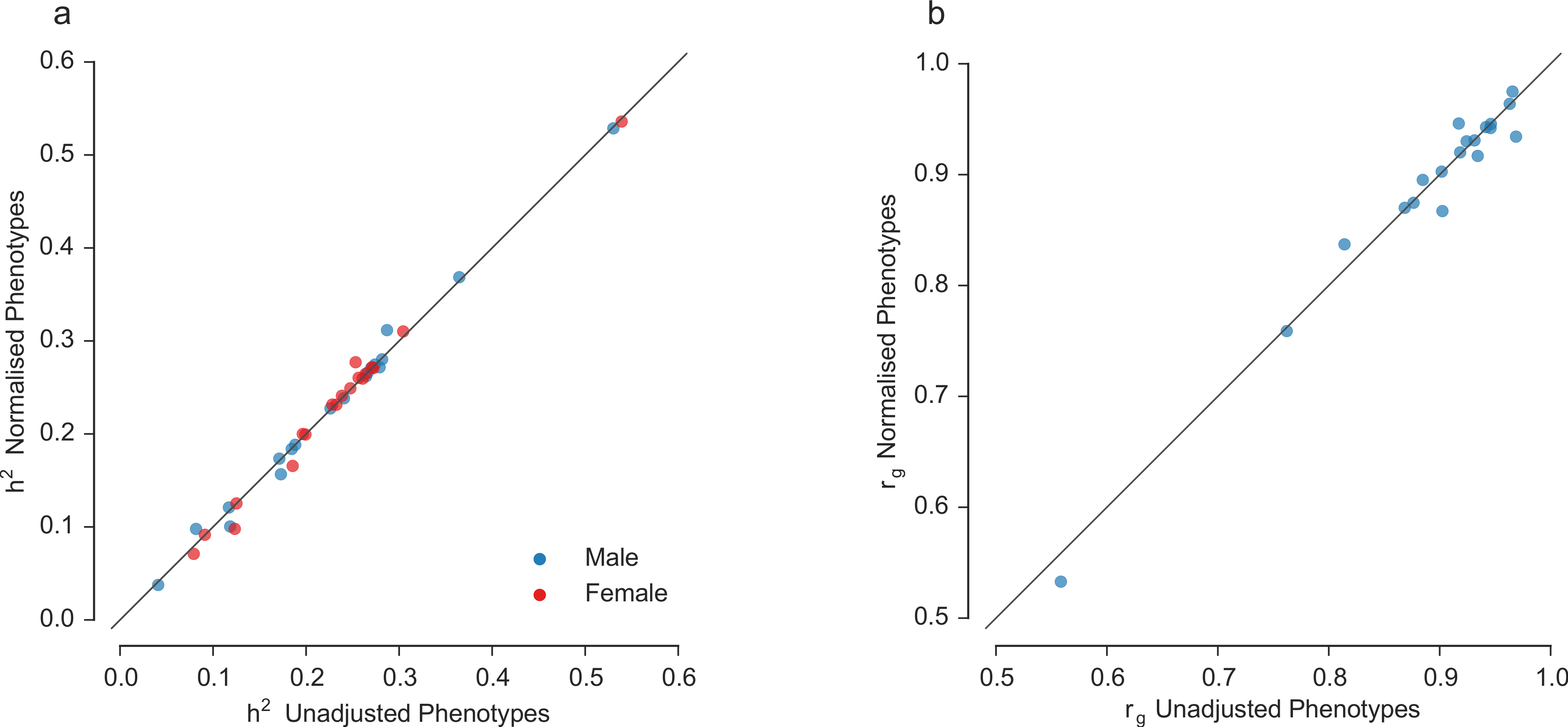
**

**Figure S4: Simulation results.** **We plot the estimates of genetic correlations (a), it’s standard error (b) and the negative** $\boldsymbol{-lo}\boldsymbol{g}_{\boldsymbol{10}}\boldsymbol{P}$**value for a test of departure from correlations equal to 1 (c) for the 10 simulated phenotypes (blue) and results of the main analysis (red) against the mean of sex specific heritability estimates.**
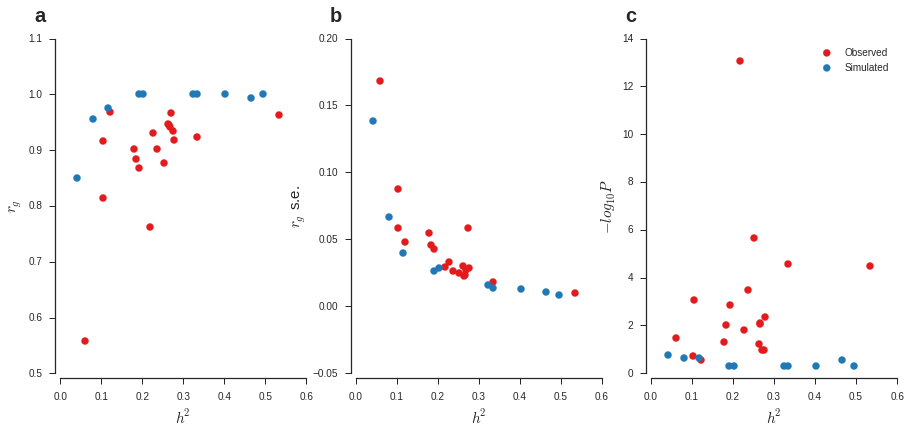


**Figure S5: Comparison of heritability and genetic correlation estimates between analyses including couples and excluding one partner from each couple on the main cohort of white-british individuals using common variants.**

**
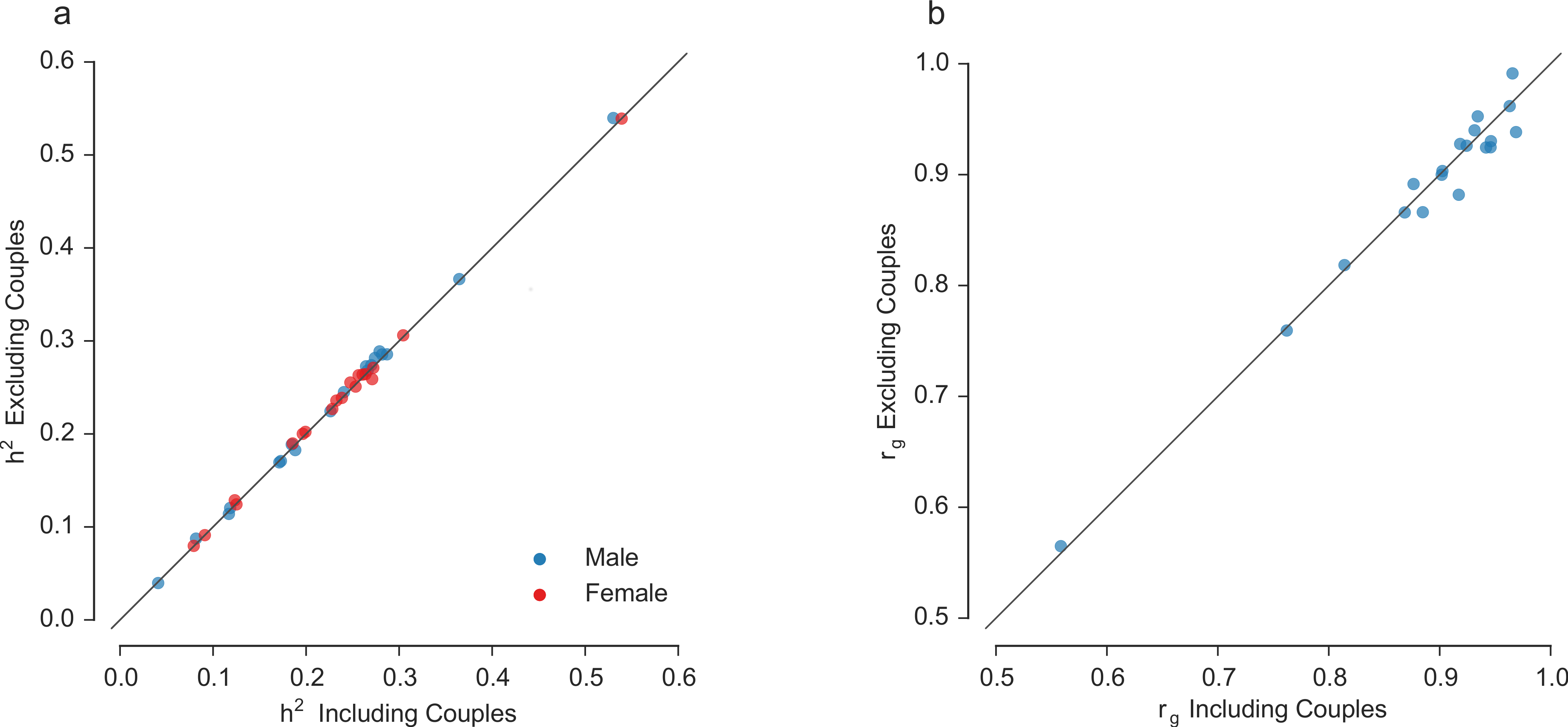
**

**Figure S6: Comparison of heritability and genetic correlation estimates between analyses covariates for population structure and additional covariates for scio-economic status on the main cohort of white-british individuals using common variants.**

**
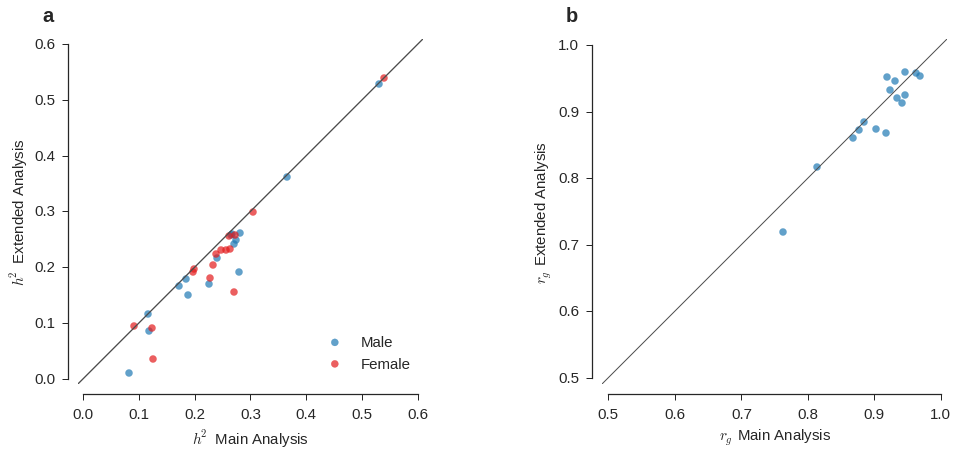
**

**Table S1: Phenotype specific sample sizes for the cohort of unrelated and related White British individuals.**

|  | **Unrelated** | | **Related** | |
| --- | --- | --- | --- | --- |
|  | **Men** | **Women** | **Men** | **Women** |
| Height | 53938 | 59716 | 9034 | 10574 |
| BMI | 53480 | 59140 | 8945 | 10453 |
| BF% | 52989 | 58912 | 8851 | 10439 |
| BMR | 52810 | 58369 | 8826 | 10338 |
| WC | 53707 | 59490 | 8983 | 10525 |
| HC | 53589 | 59198 | 8968 | 10482 |
| WHR | 53908 | 59669 | 9016 | 10561 |
| Education Age | 35726 | 41300 | 6534 | 7921 |
| FI Score | 17630 | 19190 | 2787 | 3202 |
| CF Score | 51141 | 56860 | 8515 | 10037 |
| LRS | 25330 | 44265 | 4286 | 7770 |
| Overall Health | 54040 | 59820 | 9045 | 10579 |
| BP_dia_ | 37516 | 45861 | 6214 | 8046 |
| BP_sys_ | 37422 | 45727 | 6199 | 8020 |
| FEV_1_ | 41809 | 45228 | 6930 | 7943 |
| FVC | 41809 | 45228 | 6930 | 7943 |
| PEF | 41809 | 45228 | 6930 | 7943 |
| FEV_1_/FVC | 41809 | 45228 | 6930 | 7943 |
| Pack Years | 33088 | 35731 | 5532 | 6376 |

**Table S2: Sexual dimorphism index in the unrelated White-British cohort.**

| **Name** | **SDI** |
| --- | --- |
| Height | 0.08 |
| BMI | 0.03 |
| BF% | -0.31 |
| BMR | 0.38 |
| WC | 0.15 |
| HC | 0.00 |
| WHR | 0.15 |
| Education Age | 0.00 |
| FI Score | 0.03 |
| CF Score | 1.05 |
| LRS | 0.04 |
| Overall Health | 0.04 |
| BP_dia_ | 0.05 |
| BP_sys_ | 0.05 |
| FEV_1_ | 0.38 |
| FVC | 0.39 |
| PEF | 0.43 |
| FEV_1_/FVC | -0.01 |
| Pack Years | 0.40 |

SDI = sexual dimorphism index calculated as $\frac{\mu_{m}}{\mu_{f}}-1$, where $\mu_{x}$ is the sex specific phenotypic mean.

**Table S3: Heritability and genetic correlation estimates for alternative analyses of blood pressure traits.**

|  |  | **BP_dia_** | | **BP_sys_** | |
| --- | --- | --- | --- | --- | --- |
|  |  | **Main** | **Alternative** | **Main** | **Alternative** |
| $\boldsymbol{h}_{\boldsymbol{m}}^{\boldsymbol{2}}$ | **Est.** | 0.18 | 0.13 | 0.17 | 0.12 |
|  | **S.E.** | 0.011 | 0.008 | 0.011 | 0.008 |
| $\boldsymbol{h}_{\boldsymbol{f}}^{\boldsymbol{2}}$ | **Est.** | 0.20 | 0.16 | 0.20 | 0.15 |
|  | **S.E.** | 0.009 | 0.008 | 0.009 | 0.008 |
| $\boldsymbol{r}_{\boldsymbol{g}}$ | **Est.** | 0.87 | 0.89 | 0.88 | 0.89 |
|  | **S.E.** | 0.043 | 0.045 | 0.046 | 0.049 |
| **P Value** | $\boldsymbol{r}_{\boldsymbol{g}}\boldsymbol{\neq1}$ | 0.001 | 0.013 | 0.009 | 0.021 |
|  | $\boldsymbol{h}_{\boldsymbol{m}}^{\boldsymbol{2}}\boldsymbol{\neq}\boldsymbol{h}_{\boldsymbol{f}}^{\boldsymbol{2}}$ | 0.316 | 0.002 | 0.087 | 0.008 |

Main: main analysis reported in Table 1, Alternative: Alternative analysis including all individuals and adjusting for hypertension status and medication. $h_{m}^{2}$,$h_{f}^{2}$: proportions of phenotypic variance explained by common autosomal SNPs for males and females respectively. P Value: p values from likelihood ratio test against constrained models with $r_{g}=1$ and $h_{m}^{2}=h_{f}^{2}$ respectively.

**Table S4: Heritability and genetic correlation estimates for analysis of cohort of related White-British individuals and only common variants; and unrelated White-British individuals and common and rare variants.**

|  | **Related Individuals** | | | | | | **Unrelated Common and Rare Variants** | | | | | |
| --- | --- | --- | --- | --- | --- | --- | --- | --- | --- | --- | --- | --- |
|  | $\boldsymbol{h}_{\boldsymbol{m}}^{\boldsymbol{2}}$ | | $\boldsymbol{h}_{\boldsymbol{f}}^{\boldsymbol{2}}$ | | $\boldsymbol{r}_{\boldsymbol{g}}$ | | $\boldsymbol{h}_{\boldsymbol{m}}^{\boldsymbol{2}}$ | | $\boldsymbol{h}_{\boldsymbol{f}}^{\boldsymbol{2}}$ | | $\boldsymbol{r}_{\boldsymbol{g}}$ | |
|  | **Est.** | **S.E.** | **Est.** | **S.E.** | **Est.** | **S.E.** | **Est.** | **S.E.** | **Est.** | **S.E.** | **Est.** | **S.E.** |
| Height | 0.73 | 0.03 | 0.79 | 0.03 | 0.92 | 0.04 | 0.62 | 0.01 | 0.63 | 0.01 | 0.96 | 0.01 |
| BMI | 0.32 | 0.04 | 0.36 | 0.03 | 0.94 | 0.09 | 0.32 | 0.01 | 0.30 | 0.01 | 0.95 | 0.02 |
| BF% | 0.33 | 0.04 | 0.39 | 0.03 | 0.95 | 0.08 | 0.32 | 0.01 | 0.31 | 0.01 | 0.93 | 0.03 |
| BMR | 0.51 | 0.04 | 0.42 | 0.03 | 0.89 | 0.07 | 0.43 | 0.01 | 0.36 | 0.01 | 0.91 | 0.02 |
| WC | 0.27 | 0.04 | 0.30 | 0.03 | 1.00 | 0.10 | 0.28 | 0.01 | 0.28 | 0.01 | 0.90 | 0.03 |
| HC | 0.35 | 0.04 | 0.34 | 0.03 | 0.92 | 0.09 | 0.32 | 0.01 | 0.29 | 0.01 | 0.86 | 0.03 |
| WHR | 0.21 | 0.04 | 0.30 | 0.03 | 0.77 | 0.12 | 0.23 | 0.01 | 0.30 | 0.01 | 0.74 | 0.03 |
| Ed. Age^$^ | - | - | - | - | - | - | 0.10 | 0.01 | 0.15 | 0.01 | 0.92 | 0.09 |
| FI Score | 0.29 | 0.10 | 0.40 | 0.09 | 1.00 | 0.25 | 0.33 | 0.03 | 0.35 | 0.03 | 0.91 | 0.06 |
| CI Score | 0.16 | 0.04 | 0.13 | 0.03 | 0.86 | 0.20 | 0.14 | 0.01 | 0.11 | 0.01 | 0.84 | 0.06 |
| LRS | 0.13 | 0.07 | 0.12 | 0.04 | 0.49 | 0.35 | 0.06 | 0.02 | 0.09 | 0.01 | 0.46 | 0.15 |
| OH* | 0.15 | 0.04 | 0.17 | 0.03 | 1.00 | 0.19 | 0.14 | 0.01 | 0.15 | 0.01 | 0.97 | 0.05 |
| BPdia | 0.26 | 0.05 | 0.23 | 0.04 | 0.93 | 0.14 | 0.23 | 0.01 | 0.24 | 0.01 | 0.80 | 0.04 |
| BPsys | 0.22 | 0.05 | 0.23 | 0.04 | 0.75 | 0.18 | 0.22 | 0.01 | 0.24 | 0.01 | 0.84 | 0.05 |
| FEV1 | 0.33 | 0.05 | 0.41 | 0.04 | 1.00 | 0.11 | 0.34 | 0.01 | 0.33 | 0.01 | 0.91 | 0.03 |
| FVC | 0.34 | 0.05 | 0.40 | 0.04 | 1.00 | 0.11 | 0.32 | 0.01 | 0.32 | 0.01 | 0.94 | 0.03 |
| PEF | 0.33 | 0.05 | 0.34 | 0.04 | 0.70 | 0.11 | 0.27 | 0.01 | 0.28 | 0.01 | 0.93 | 0.04 |
| FEV1/FVC | 0.32 | 0.05 | 0.26 | 0.04 | 1.00 | 0.13 | 0.35 | 0.01 | 0.30 | 0.01 | 0.97 | 0.03 |
| PY^†^ | 0.19 | 0.06 | 0.36 | 0.05 | 0.91 | 0.21 | 0.21 | 0.02 | 0.23 | 0.01 | 0.89 | 0.06 |

*Overall Health, ^†^Pack Years. $h_{m}^{2}$,$h_{f}^{2}$= proportions of phenotypic variance explained by common autosomal SNPs for males and females respectively. ^$^Analysis for Ed. Age failed to converge for the cohort of related individuals.

**Table S5: Heritability and genetic correlation estimates for analysis of categorical traits with matched cohorts.**

|  | **Number** | $\boldsymbol{h}_{\boldsymbol{m}}^{\boldsymbol{2}}$ | | $\boldsymbol{h}_{\boldsymbol{f}}^{\boldsymbol{2}}$ | | $\boldsymbol{r}_{\boldsymbol{g}}$ | | **P Value** | |
| --- | --- | --- | --- | --- | --- | --- | --- | --- | --- |
|  |  | **Est.** | **S.E.** | **Est.** | **S.E.** | **Est.** | **S.E** | $\boldsymbol{r}_{\boldsymbol{g}}\boldsymbol{\neq1}$ | $\boldsymbol{h}_{\boldsymbol{m}}^{\boldsymbol{2}}\boldsymbol{\neq}\boldsymbol{h}_{\boldsymbol{f}}^{\boldsymbol{2}}$ |
| Education Age | 34785 | 0.09 | 0.011 | 0.14 | 0.012 | 0.84 | 0.09 | 0.04 | 0.01 |
| FI Score | 17121 | 0.24 | 0.022 | 0.26 | 0.022 | 0.97 | 0.06 | 0.28 | 0.54 |
| LRS | 25309 | 0.04 | 0.014 | 0.09 | 0.016 | 0.57 | 0.20 | 0.04 | 0.06 |
| Overall Health | 52870 | 0.11 | 0.008 | 0.13 | 0.008 | 0.98 | 0.05 | 0.34 | 0.20 |

Number: Number of samples for each sex after matching, i.e., total number of samples used in the analysis corresponds to twice the given number. $h_{m}^{2}$,$h_{f}^{2}$: proportions of phenotypic variance explained by common autosomal SNPs for males and females respectively. P Value: p values from likelihood ratio test against constrained models with $r_{g}=1$ and $h_{m}^{2}=h_{f}^{2}$ respectively.

**Table S6: Comparison of results for departure of genetic correlations from one and differences in heritability between sexes across different analyses.**

|  | **P value** $\boldsymbol{r}_{\boldsymbol{g}}\boldsymbol{\neq1}$ | | | |  | **P Value** $\boldsymbol{h}_{\boldsymbol{m}}^{\boldsymbol{2}}\boldsymbol{\neq}\boldsymbol{h}_{\boldsymbol{f}}^{\boldsymbol{2}}$ | | | |
| --- | --- | --- | --- | --- | --- | --- | --- | --- | --- |
|  | **Main** | **Norm.** | **Cov.** | **Couples** |  | **Main** | **Norm.** | **Cov.** | **Couples** |
| **Height** | *3*×10^-5^ | *5*×10^-5^ | *3*×10^-6^ | *9*×10^-5^ |  | 0.343 | 0.436 | 0.256 | 0.952 |
| **BMI** | *0.008* | *0.005* | *0.001* | *0.002* |  | 0.110 | 0.194 | 0.112 | 0.124 |
| **BF%** | *0.009* | *0.010* | *0.001* | *0.003* |  | 0.575 | 0.549 | 0.425 | 0.427 |
| **BMR** | *2*×10^-5^ | *7*×10^-5^ | *1*×10^-4^ | *2*×10^-4^ |  | *3*×10^-8^ | *8*×10^-8^ | *1*×10^-8^ | *4*×10^-7^ |
| **WC** | *3*×10^-4^ | *3*×10^-4^ | *5*×10^-5^ | *0.001* |  | 0.462 | 0.535 | 0.243 | 0.453 |
| **HC** | *2*×10^-6^ | *1*×10^-6^ | *3*×10^-6^ | *1*×10^-4^ |  | *0.012* | *0.023* | *0.001* | *0.015* |
| **WHR** | *9*×10^-14^ | *3*×10^-14^ | *1*×10^-13^ | *7*×10^-12^ |  | *1*×10^-7^ | *7*×10^-8^ | *7*×10^-13^ | *3*×10^-9^ |
| **Ed. Age** | 0.186 | 0.233 | 0.425 | 0.106 |  | *0.004* | 0.071 | 0.090 | *0.026* |
| **FI Score** | 0.106 | 0.073 | 0.191 | 0.205 |  | 0.794 | 0.996 | 0.247 | 0.372 |
| **CF Score** | *0.001* | *0.003* | *0.001* | *0.003* |  | *0.019* | *0.008* | *0.054* | 0.057 |
| **LRS** | *0.032* | *0.043* | *-* | 0.054 |  | *0.028* | 0.056 | - | *0.037* |
| **Overall Health** | 0.267 | 0.134 | 0.268 | 0.120 |  | 0.641 | 0.806 | 0.640 | 0.478 |
| **BP_dia_** | *0.001* | *0.002* | *0.001* | *0.002* |  | 0.316 | 0.289 | 0.220 | 0.390 |
| **BP_sys_** | *0.009* | *0.016* | *0.010* | *0.006* |  | 0.087 | 0.072 | 0.081 | 0.052 |
| **FEV_1_** | *0.004* | *0.005* | *0.085* | *0.017* |  | 0.488 | 0.488 | 0.824 | 0.330 |
| **FVC** | 0.058 | 0.058 | 0.125 | *0.028* |  | 0.786 | 0.853 | 0.930 | 0.545 |
| **PEF** | *0.015* | *0.014* | *0.075* | *0.042* |  | 0.885 | 0.765 | 0.437 | 0.867 |
| **FEV_1_/FVC** | 0.107 | 0.144 | - | 0.394 |  | *0.013* | *0.011* | *-* | *0.017* |
| **Pack Years** | *0.049* | *0.024* | *-* | 0.057 |  | 0.461 | 0.605 | - | 0.293 |

Values with a light green background are nominally significant at P < 0.05, values with a dark green background are significant after Bonf. adjustment. Main: results reported in the main manuscript; Norm.: results on rank normalised phenotypes; Cov.: results with additional covariates adjusting for socio-economic status, health status and educational attainment; Couples: analysis without spouse pairs.

**Table S7: Heritability and genetic correlation estimates for analysis of rank normalised traits in unrelated White-British.**

|  | $\boldsymbol{h}_{\boldsymbol{m}}^{\boldsymbol{2}}$ | | $\boldsymbol{h}_{\boldsymbol{f}}^{\boldsymbol{2}}$ | | $\boldsymbol{r}_{\boldsymbol{g}}$ | | **P Value** | |
| --- | --- | --- | --- | --- | --- | --- | --- | --- |
|  | **Est.** | **S.E.** | **Est.** | **S.E.** | **Est.** | **S.E.** | $\boldsymbol{r}_{\boldsymbol{g}}\boldsymbol{\neq1}$ | $\boldsymbol{h}_{\boldsymbol{m}}^{\boldsymbol{2}}\boldsymbol{\neq}\boldsymbol{h}_{\boldsymbol{f}}^{\boldsymbol{2}}$ |
| Height | 0.53 | 0.007 | 0.54 | 0.007 | 0.96 | 0.010 | 5×10^-5^ | 0.44 |
| BMI | 0.27 | 0.008 | 0.26 | 0.008 | 0.94 | 0.022 | 4×10^-3^ | 0.19 |
| BF% | 0.27 | 0.008 | 0.26 | 0.008 | 0.94 | 0.023 | 0.01 | 0.55 |
| BMR | 0.37 | 0.008 | 0.31 | 0.008 | 0.93 | 0.018 | 7×10^-5^ | 8×10^-8^ |
| WC | 0.24 | 0.008 | 0.23 | 0.008 | 0.90 | 0.027 | 3×10^-4^ | 0.53 |
| HC | 0.27 | 0.008 | 0.24 | 0.008 | 0.87 | 0.025 | 1×10^-6^ | 0.02 |
| WHR | 0.19 | 0.008 | 0.25 | 0.008 | 0.76 | 0.029 | 3×10^-14^ | 7×10^-8^ |
| Education Age | 0.1 | 0.011 | 0.13 | 0.010 | 0.95 | 0.076 | 0.23 | 0.07 |
| FI Score | 0.27 | 0.022 | 0.27 | 0.021 | 0.92 | 0.062 | 0.07 | 1.00 |
| CF Score | 0.12 | 0.008 | 0.09 | 0.007 | 0.84 | 0.058 | 2×10^-3^ | 0.01 |
| LRS | 0.04 | 0.014 | 0.07 | 0.009 | 0.53 | 0.184 | 0.04 | 0.06 |
| Overall Health | 0.1 | 0.008 | 0.10 | 0.007 | 0.93 | 0.058 | 0.13 | 0.81 |
| BP_dia_ | 0.18 | 0.011 | 0.20 | 0.009 | 0.87 | 0.043 | 1×10^-3^ | 0.29 |
| BP_sys_ | 0.17 | 0.011 | 0.20 | 0.009 | 0.90 | 0.045 | 0.02 | 0.07 |
| FEV_1_ | 0.28 | 0.010 | 0.27 | 0.010 | 0.92 | 0.029 | 0.01 | 0.49 |
| FVC | 0.26 | 0.010 | 0.26 | 0.009 | 0.95 | 0.031 | 0.06 | 0.85 |
| PEF | 0.23 | 0.010 | 0.23 | 0.009 | 0.93 | 0.033 | 0.01 | 0.76 |
| FEV_1_/FVC | 0.31 | 0.010 | 0.28 | 0.009 | 0.97 | 0.024 | 0.14 | 0.01 |
| Pack Years | 0.16 | 0.012 | 0.17 | 0.012 | 0.87 | 0.062 | 0.02 | 0.61 |

**Table S8: Parameter estimates for the 10 simulated phenotypes.**

|  | $\boldsymbol{h}_{\boldsymbol{m}}$ | $\boldsymbol{h}_{\boldsymbol{f}}$ | $\boldsymbol{r}_{\boldsymbol{g}}$ | $\boldsymbol{r}_{\boldsymbol{g}}$ **s.e.** | **P** $\boldsymbol{r}_{\boldsymbol{g}}\boldsymbol{\neq1}$ |
| --- | --- | --- | --- | --- | --- |
| **1** | 0.04 | 0.05 | 0.85 | 0.138 | 0.17 |
| **2** | 0.07 | 0.09 | 0.96 | 0.067 | 0.24 |
| **3** | 0.11 | 0.12 | 0.98 | 0.040 | 0.23 |
| **5** | 0.18 | 0.22 | 1.00 | 0.029 | 0.50 |
| **4** | 0.19 | 0.19 | 1.00 | 0.027 | 0.50 |
| **6** | 0.31 | 0.34 | 1.00 | 0.016 | 0.50 |
| **7** | 0.37 | 0.30 | 1.00 | 0.014 | 0.50 |
| **8** | 0.40 | 0.53 | 0.99 | 0.011 | 0.29 |
| **9** | 0.46 | 0.35 | 1.00 | 0.013 | 0.50 |
| **10** | 0.51 | 0.48 | 1.00 | 0.008 | 0.50 |

**Table S9: Heritability and genetic correlation estimates for analysis of unrelated White-British without couples.**

|  | $\boldsymbol{h}_{\boldsymbol{m}}^{\boldsymbol{2}}$ | | $\boldsymbol{h}_{\boldsymbol{f}}^{\boldsymbol{2}}$ | | $\boldsymbol{r}_{\boldsymbol{g}}$ | | **P Value** | |
| --- | --- | --- | --- | --- | --- | --- | --- | --- |
|  | **Est.** | **S.E.** | **Est.** | **S.E.** | **Est.** | **S.E.** | $\boldsymbol{r}_{\boldsymbol{g}}\boldsymbol{\neq1}$ | $\boldsymbol{h}_{\boldsymbol{m}}^{\boldsymbol{2}}\boldsymbol{\neq}\boldsymbol{h}_{\boldsymbol{f}}^{\boldsymbol{2}}$ |
| Height | 0.54 | 0.008 | 0.54 | 0.007 | 0.96 | 0.01 | 9×10^-5^ | 0.95 |
| BMI | 0.28 | 0.009 | 0.26 | 0.008 | 0.92 | 0.02 | 1×10^-3^ | 0.12 |
| BF% | 0.27 | 0.009 | 0.26 | 0.008 | 0.92 | 0.03 | 3×10^-3^ | 0.43 |
| BMR | 0.37 | 0.009 | 0.31 | 0.008 | 0.93 | 0.02 | 2×10^-4^ | 4×10^-7^ |
| WC | 0.24 | 0.009 | 0.24 | 0.008 | 0.90 | 0.03 | 6×10^-4^ | 0.45 |
| HC | 0.27 | 0.009 | 0.24 | 0.008 | 0.89 | 0.03 | 1×10^-4^ | 0.01 |
| WHR | 0.18 | 0.009 | 0.26 | 0.008 | 0.76 | 0.03 | 7×10^-12^ | 3×10^-9^ |
| Education Age | 0.09 | 0.012 | 0.12 | 0.011 | 0.88 | 0.09 | 0.11 | 0.03 |
| FI Score | 0.29 | 0.024 | 0.26 | 0.022 | 0.95 | 0.06 | 0.20 | 0.37 |
| CF Score | 0.11 | 0.009 | 0.09 | 0.008 | 0.82 | 0.06 | 2×10^-3^ | 0.06 |
| LRS | 0.04 | 0.016 | 0.08 | 0.010 | 0.56 | 0.19 | 0.05 | 0.04 |
| Overall Health | 0.12 | 0.008 | 0.13 | 0.008 | 0.94 | 0.05 | 0.12 | 0.48 |
| BP_dia_ | 0.19 | 0.012 | 0.20 | 0.010 | 0.87 | 0.05 | 1×10^-3^ | 0.39 |
| BP_sys_ | 0.17 | 0.012 | 0.20 | 0.010 | 0.87 | 0.05 | 0.01 | 0.05 |
| FEV_1_ | 0.29 | 0.011 | 0.27 | 0.010 | 0.93 | 0.03 | 0.02 | 0.33 |
| FVC | 0.27 | 0.011 | 0.26 | 0.010 | 0.93 | 0.03 | 0.03 | 0.54 |
| PEF | 0.22 | 0.011 | 0.23 | 0.010 | 0.94 | 0.04 | 0.04 | 0.87 |
| FEV_1_/FVC | 0.29 | 0.011 | 0.25 | 0.010 | 0.99 | 0.03 | 0.39 | 0.02 |
| Pack Years | 0.17 | 0.013 | 0.19 | 0.012 | 0.90 | 0.06 | 0.06 | 0.29 |

**Table S10: Heritability and genetic correlation estimates for analysis including additional covariates for socio-economic status.**

|  | $\boldsymbol{h}_{\boldsymbol{m}}^{\boldsymbol{2}}$ | | $\boldsymbol{h}_{\boldsymbol{f}}^{\boldsymbol{2}}$ | | $\boldsymbol{r}_{\boldsymbol{g}}$ | | **P Value** | |
| --- | --- | --- | --- | --- | --- | --- | --- | --- |
|  | **Est.** | **S.E.** | **Est.** | **S.E.** | **Est.** | **S.E.** | $\boldsymbol{r}_{\boldsymbol{g}}\boldsymbol{\neq1}$ | $\boldsymbol{h}_{\boldsymbol{m}}^{\boldsymbol{2}}\boldsymbol{\neq}\boldsymbol{h}_{\boldsymbol{f}}^{\boldsymbol{2}}$ |
| Height | 0.53 | 0.007 | 0.54 | 0.007 | 0.96 | 0.010 | 3×10^-6^ | 0.26 |
| BMI | 0.25 | 0.008 | 0.23 | 0.008 | 0.92 | 0.025 | 1×10^-3^ | 0.11 |
| BF% | 0.24 | 0.008 | 0.23 | 0.008 | 0.91 | 0.026 | 9×10^-4^ | 0.43 |
| BMR | 0.36 | 0.008 | 0.30 | 0.008 | 0.93 | 0.018 | 1×10^-4^ | 0.00 |
| WC | 0.22 | 0.008 | 0.20 | 0.008 | 0.87 | 0.030 | 5×10^-5^ | 0.24 |
| HC | 0.26 | 0.008 | 0.22 | 0.008 | 0.87 | 0.026 | 3×10^-6^ | 1×10^-3^ |
| WHR | 0.15 | 0.008 | 0.23 | 0.008 | 0.72 | 0.034 | 1×10^-13^ | 7×10^-13^ |
| Education Age | 0.01 | 0.010 | 0.03 | 0.009 | 0.87 | 0.538 | 0.42 | 0.09 |
| FI Score | 0.19 | 0.022 | 0.16 | 0.020 | 0.92 | 0.096 | 0.19 | 0.25 |
| CF Score | 0.12 | 0.008 | 0.09 | 0.007 | 0.82 | 0.058 | 8×10^-4^ | 0.05 |
| LRS^$^ | - | - | - | - | - | - | - | - |
| Overall Health | 0.09 | 0.008 | 0.09 | 0.007 | 0.95 | 0.068 | 0.27 | 0.64 |
| BP_dia_ | 0.18 | 0.011 | 0.20 | 0.009 | 0.86 | 0.044 | 8×10^-4^ | 0.22 |
| BP_sys_ | 0.17 | 0.011 | 0.19 | 0.009 | 0.88 | 0.047 | 0.01 | 0.08 |
| FEV_1_ | 0.26 | 0.010 | 0.26 | 0.010 | 0.95 | 0.031 | 0.08 | 0.82 |
| FVC | 0.26 | 0.010 | 0.26 | 0.009 | 0.96 | 0.031 | 0.12 | 0.93 |
| PEF | 0.17 | 0.010 | 0.18 | 0.009 | 0.95 | 0.042 | 0.08 | 0.44 |
| FEV_1_/FVC^$^ | - | - | - | - | - | - | - | - |
| Pack Years^$^ | - | - | - | - | - | - | - | - |

^$^ Not converged

**Table S11: Prediction accuracies for bi- and univariate models**

|  | **Accuracy** | | **Improvement** |
| --- | --- | --- | --- |
|  | **Bivariate** | **Univariate** |  |
| **Height** | 0.4993 | 0.4984 | 0.0019 |
| **BMI** | 0.2539 | 0.2522 | 0.0066 |
| **BF%** | 0.2587 | 0.2558 | 0.0112 |
| **BMR** | 0.3209 | 0.3078 | 0.0424 |
| **WC** | 0.2340 | 0.2341 | -0.0004 |
| **HC** | 0.2476 | 0.2454 | 0.0091 |
| **WHR** | 0.2053 | 0.2004 | 0.0243 |
| Education Age | 0.0841 | 0.0810 | 0.0389 |
| FI Score | 0.1304 | 0.1318 | -0.0102 |
| CF Score | 0.1009 | 0.1023 | -0.0132 |
| LRS | 0.0224 | 0.0239 | -0.0625 |
| Overall Health | 0.1341 | 0.1332 | 0.0067 |
| BP_dia_ | 0.1688 | 0.1693 | -0.0029 |
| BP_sys_ | 0.1617 | 0.1605 | 0.0072 |
| **FEV_1_** | 0.2471 | 0.2413 | 0.0239 |
| FVC | 0.2383 | 0.2332 | 0.0218 |
| **PEF** | 0.2141 | 0.2097 | 0.0208 |
| **FEV_1_/FVC** | 0.2562 | 0.2558 | 0.0018 |
| Pack Years | 0.1428 | 0.1393 | 0.0251 |

Phenotypes in bold show evidence of genetic heterogeneity between sexes (P < 0.05 for r_g_ ≠ 1 or $h_{m}^{2} \neq h_{f}^{2}$) and substantial heritability ($h_{m}^{2}>0.2$ and $h_{f}^{2}>0.2$). Accuracy: correlation between predicted additive genetic value and observed phenotype adjusted for fixed effects. Improvement: relative improvement in accuracy of the bivariate model to the univariate model, i.e., ration of accuracy bivariate and univariate minus one.
